# Supplementary material for: Effects of Trichoderma harzianum combined with Phanerochaete chrysosporium on lignin degradation and humification during chicken manure and rice husk composting
Source: Front Microbiol. 2025 Feb 28;16:1515931. doi: 10.3389/fmicb.2025.1515931 (PMC11906335; doi:10.3389/fmicb.2025.1515931)
Supplement: Supplementary file 1 [file Data_Sheet_1.zip › 6.Function/HF_BFF/FunGuild/l2/BFF_vs_HF_STAMP.pdf]

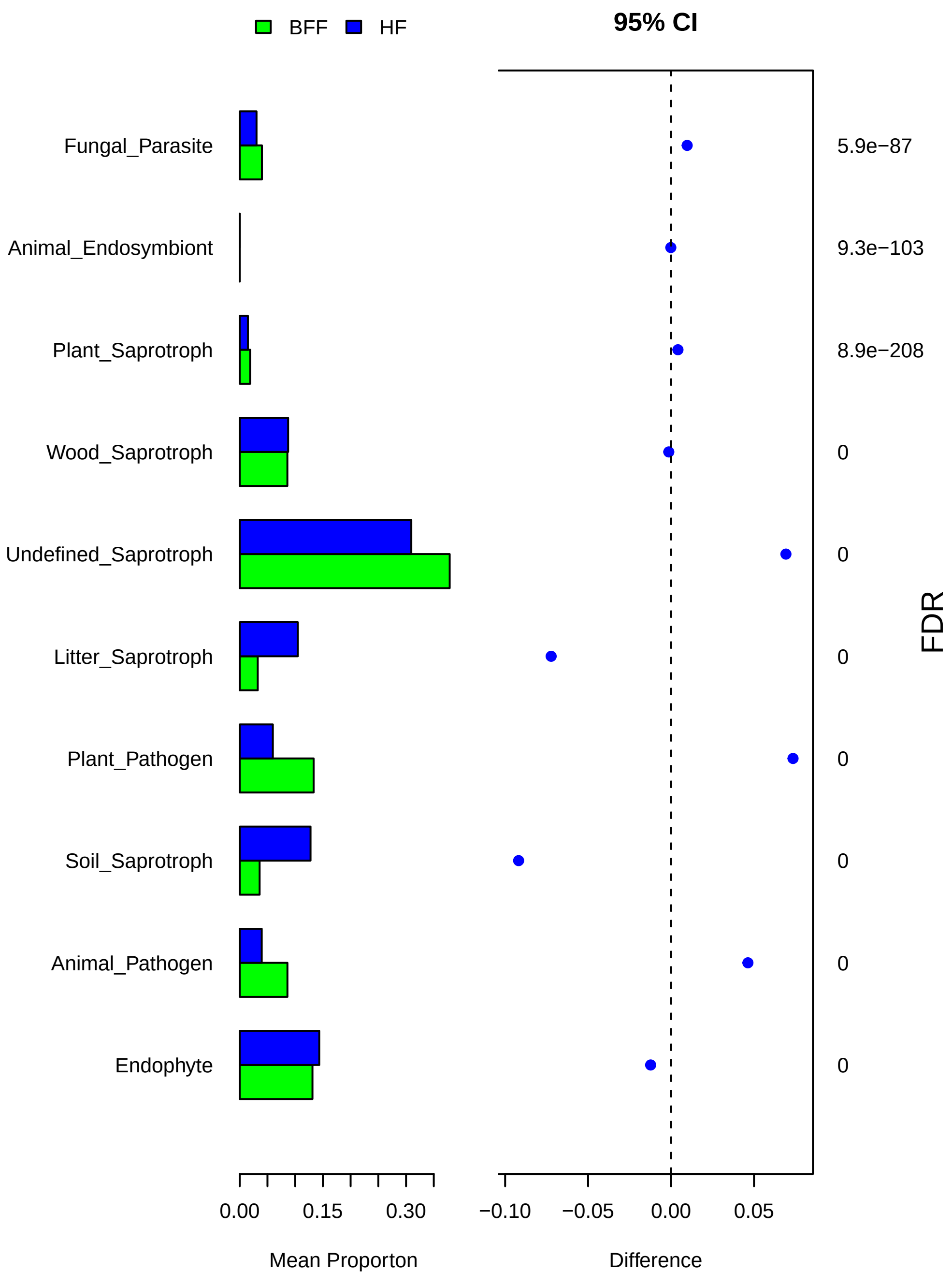

BFF
HF
95% CI
Fungal_Parasite
Animal_Endosymbiont
Plant_Saprotroph
Wood_Saprotroph
Undefined_Saprotroph
Litter_Saprotroph
Plant_Pathogen
Soil_Saprotroph
Animal_Pathogen
Endophyte
5.9e−87
9.3e−103
8.9e−208
0
0
0
0
0
0
0
FDR
0.00
0.15
0.30
−0.10
−0.05
0.00
0.05
Mean Proporton
Difference
